# Genome-Wide Association Study Reveals Growth-Related SNPs and Candidate Genes in Largemouth Bass (*Micropterus salmoides*) Adapted to Hypertonic Environments

**DOI:** 10.3390/ijms26051834

**Published:** 2025-02-20

**Authors:** Miaomiao Ding, Yifan Tao, Jixiang Hua, Yalun Dong, Siqi Lu, Jun Qiang, Jixiang He

**Affiliations:** 1 Wuxi Fisheries College, Nanjing Agricultural University, Wuxi 214081, China; d2490443127@163.com (M.D.); huajixiang@foxmail.com (J.H.); 2 Key Laboratory of Freshwater Fisheries and Germplasm Resources Utilization, Ministry of Agriculture and Rural Affairs, Freshwater Fisheries Research Center, Chinese Academy of Fishery Sciences, Wuxi 214081, China; taoyifan@ffrc.cn (Y.T.); dongyalun@ffrc.cn (Y.D.); lusiqi@ffrc.cn (S.L.); 3 Fisheries Research Institute, Anhui Academy of Agricultural Sciences, Hefei 230041, China

**Keywords:** *Micropterus salmoides*, salinity stress, growth traits, GWAS

## Abstract

Sustainable development of the largemouth bass industry is hindered by limited freshwater resources. Consequently, the expansion of farming space by brackish and saline water aquaculture has become imperative. Largemouth bass is an economically important freshwater fish species. However, there is presently a lack of germplasm resources with the capacity to adapt to hypertonic environments and maintain rapid growth. A genome-wide association study is a technique used for the detection of genetic variants associated with specific phenotypic traits. In this study, we firstly applied this technique to explore the potential single-nucleotide polymorphism (SNP) locus and candidate genes associated with rapid growth and adaptation to the hypertonic environment of largemouth bass. A total of 10 potential growth-related SNPs were obtained on chromosome 16, and SNP16:4120214 was a significant SNP peak. Based on these SNPs, 23 candidate genes were annotated in the genome, including *Nkcc1*, *Mapkap1*, *Hmgcs1*, *Slc27a6,* and *Shroom3*. *Shroom3* expression was significantly higher in individuals enriched for the most growth-advantageous genotypes. *Shroom3* upregulation is beneficial for fish growth in hyperosmotic environments. This study provides insight into the genetic basis of rapid growth in hypertonic environments and foundational information for the future breeding of salt-tolerant largemouth bass.

## 1. Introduction

Salinity is a stressor that primarily affects the osmotic pressure equilibrium on the inner and outer fish body. Euryhaline fishes can mitigate the effects of salinity fluctuations through the gill and kidney, which are part of the osmoregulatory system [1]. The capacity for adaptation to salinity is subject to great variation among different freshwater fish species. Previous research has illustrated that mandarin fish (*Siniperca chuatsi*) exhibited better growth performance at 5‰ salinity compared with freshwater environments [2]. Grass carp (*Ctenopharyngodon idellus*) growth was not significantly impacted when salinity levels were below 5‰, whereas a negative effect was observed when salinity levels exceeded 5‰ [3]. Nile tilapia (*Oreochromis niloticus*) exhibit a capacity for tolerance to salinities of up to 16‰, but their growth is inhibited by salinities above 20‰ [4,5]. A negative correlation has been demonstrated between increased salinity and the growth performance of catfish (*Lophiosilurus alexandri*) [6]. Therefore, it is essential to understand the genetic mechanism by which salinity change affects fish growth to provide foundational information for brackish aquaculture development. Expanding freshwater aquaculture into brackish and saline waters has become important because of the shortage of freshwater resources [7,8]. Therefore, understanding the genetic foundation of fish growth in salt water can enhance breeding value and advance aquaculture.

Genome-wide association studies (GWASs) using high-throughput genotyping data are effective for screening significant variant loci associated with complex traits. The GWAS advantages of a wide screening range and high accuracy have led to its popularity in the study of fish traits, including sex identification [9], stress resistance [10], and disease resistance [11]. This technique has been employed to identify growth-related genes in some fish species, including the rainbow trout (*Oncorhynchus mykiss*) [12], turbot (*Scophthalmus maximus*) [13], and carp (*Cyprinus carpio*) [14]. Furthermore, GWAS has been applied to the selection of salt-tolerant fish. A total of 11 significant single-nucleotide polymorphisms (SNPs) together with quantitative trait locus (QTL) regions and candidate genes significantly associated with salt tolerance traits in a genetically improved farmed tilapia population were discovered through GWAS [15]. Through QTL mapping and GWAS in a hybrid F_2_ family of Mozambique and Nile tilapia, two large-effect QTLs for salt tolerance were identified [16]. These results demonstrated that GWAS is an effective technique for the selection of economically advantageous traits in fish.

The largemouth bass (*Micropterus salmoides*) is a carnivorous fish with high nutritional value. Its fast growth, absence of intermuscular spines, strong adaptability, and short culture cycle have contributed to its popularity with the market and consumers. The largemouth bass is primarily cultivated in freshwater, but a shortage of freshwater resources hinders the development of the largemouth bass industry. Studies have demonstrated that largemouth bass can maintain a high survival rate at salinities below 10‰, but excessive salinity can result in tissue and organ lesions, and alterations to the intestinal microbial community [17]. In addition, a suitable salinity environment can promote growth and improve the flesh quality of largemouth bass [18,19]. The detailed mechanisms by which hypertonic environments affect largemouth bass growth remain unclear.

Consequently, in this study, GWAS was used to identify growth loci in largemouth bass under hyperosmotic stress. Following the gene targeting of SNP loci, the functions and pathways of affected genes were analyzed. Subsequently, next-generation sequencing (NGS) was conducted to validate and further explore the significant SNP peak. Ultimately, *Shroom3* was identified as a crucial gene regulating fish growth in hyperosmotic environments. Moreover, the *Shroom3* expression patterns were compared between individuals with different numbers of growth-advantageous genotypes. This study offers new insights into breeding salt-tolerant largemouth bass with enhanced growth and promotes the sustainable development of largemouth bass aquaculture.

## 2. Results

### 2.1. Sequencing Data Statistics and Population Structure Analysis

**Figure 1:**
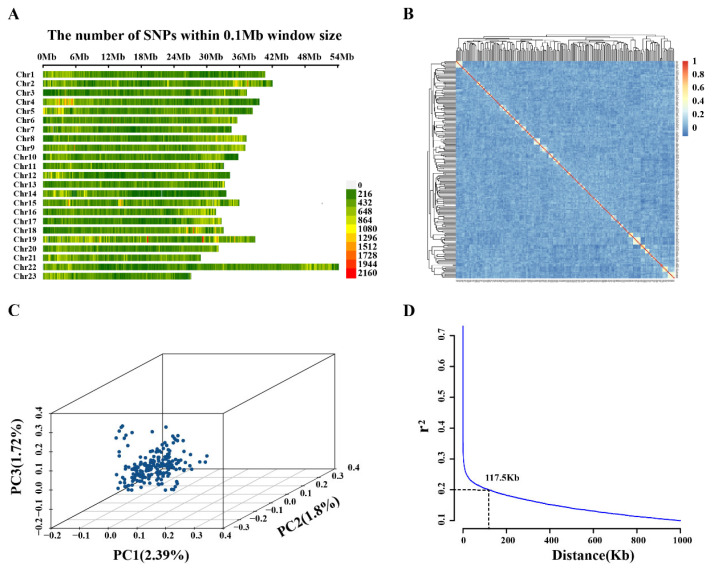
Sequencing data and population genetic variation. (**A**) Largemouth bass chromosomal density and distribution of high-quality SNPs. (**B**) Principal component analysis of the experimental population. (**C**) G-matrix of inter-individual kinship. (**D**) Genome-wide average LD decay map.

There were a total of 200 largemouth bass used for whole-genome resequencing. The total number of raw reads obtained was 8,310,639,878, with each individual obtaining an average of 41,553,199.39 ± 5,704,786.73 raw reads. Among these, the proportion of high-quality bases (Q > 30) was as high as 96%, and the average GC content was 40.78%. These reads were aligned to the largemouth bass reference genome, with a more than 99% coverage rate. Low-quality reads were removed from the raw data, resulting in 8,100,363,888 clean reads (Supplementary Table S3). After genotyping, screening of raw SNPs resulted in 3,968,611 high-quality SNPs on 23 chromosomes, which were used for GWAS. The genome-wide average SNP density was 5 SNPs/kb, with the highest coverage on chromosome 22 and the lowest coverage on chromosome 23 (Figure 1A).

PCA of the experimental populations revealed no apparent stratification, and the genetic correlation between individuals was relatively weak (Figure 1B,C). These results indicated that the largemouth bass population used in this study can be considered a natural population with a rich genetic background, which is ideal for GWAS. When the neighboring SNP distance exceeded 100 kb, the LD value decayed to below 0.2 (Figure 1D). The largemouth bass genome size is 844.9 Mb [20]; therefore, the use of 3,968,611 SNPs in this study was considered sufficient to cover the genetic variation within the population.

### 2.2. Statistical Analysis of Growth Data for GWAS and Heritability Assessment of Growth Traits

**Figure 2:**
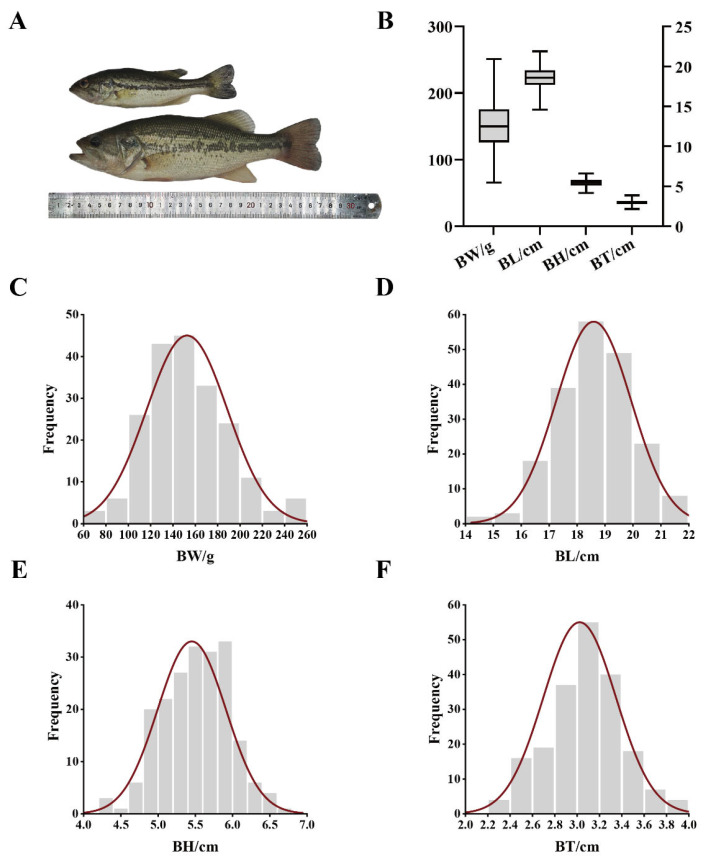
Phenotypic data of the largemouth bass population. (**A**) Comparison of individuals with extreme body sizes from the same batch. (**B**) Statistics of the growth traits across all individuals in analysis populations. Frequency distributions are shown for (**C**) body weight (BW), (**D**) body length (BL), (**E**) body height (BH), and (**F**) body thickness (BT).

The BW, BL, BH, and BT of 200 measured largemouth bass had average values of 152.56 ± 36.14 g, 18.59 ± 1.35 cm, 5.45 ± 0.46 cm, and 3.03 ± 0.32 cm, respectively. Significant inter-individual variation was observed in growth phenotypes, and BW exhibited the highest degree of phenotypic variance, with a coefficient of variation of 23.69%. BL had the smallest coefficient of variation at 7.26%. Additionally, every trait approximated a normal distribution (Figure 2, Supplementary Table S4).

This study found that BL, BW, BH, and BT had moderate to high heritability (0.468–0.539, and BT had the lowest heritability (0.468). These four traits showed strong genetic correlations (0.880–0.990) (Supplementary Table S5).

### 2.3. Screening of Growth-Associated SNPs in Hypertonic Environments

**Figure 3:**
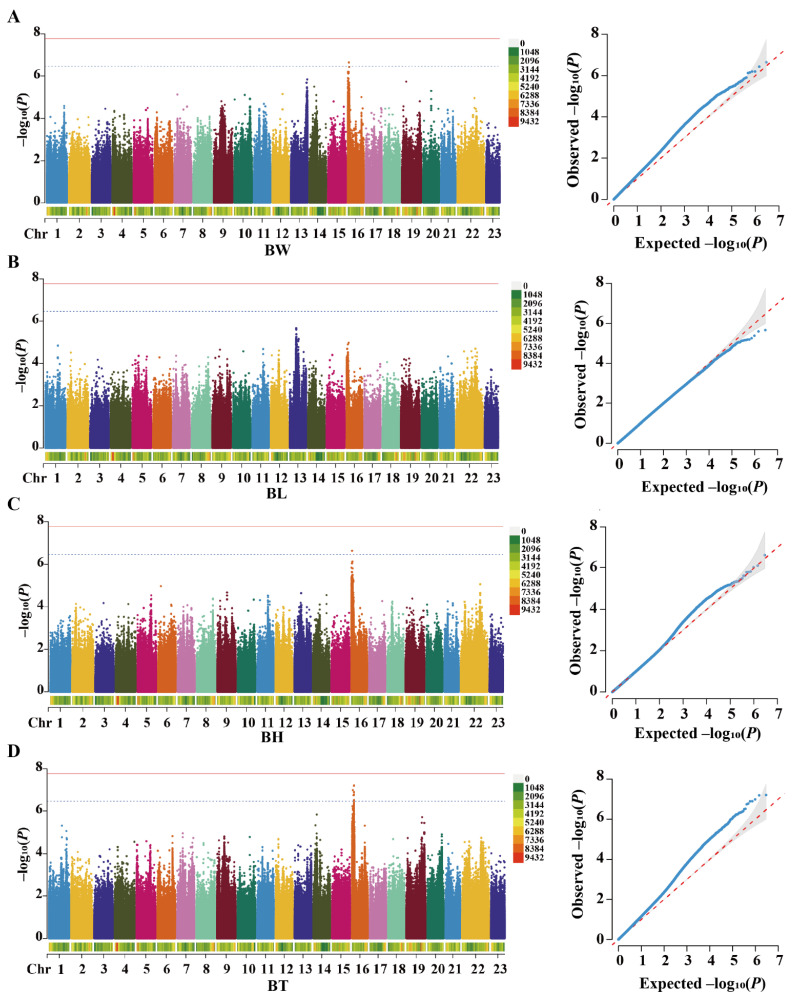
Manhattan plots (left) and quantile–quantile plots (right) of growth traits in GWAS analysis for (**A**) body height (BH), (**B**) body thickness (BT), (**C**) body weight (BW), and (**D**) body length (BL). In the Manhattan plots, the solid lines indicate the threshold *p* value for genome-wide significance association and the dashed lines indicate the threshold *p* value for suggestive association.

A total of 10 growth-associated SNPs were found on chromosome 16, as shown in the Manhattan plot (Figure 3). There was one SNP each associated with BH and BW, nine SNPs associated with BT, and no SNP associated with BL. SNP16:1462643 was associated with both BH and BT. SNP16:3984271 mutations in the exon area were synonymous mutations, and the remaining nine SNPs were located in the non-coding region of the gene. SNP16:4120214 is a significant SNP peak on chr16. Details of all SNPs are in Table 1.

### 2.4. Candidate Gene Identification and Functional Annotation

A total of 23 genes were identified within the 100 kb region upstream and downstream of the 10 growth-related SNPs. Three genes were associated with both BH and BT, fifteen genes were associated with BT, and five genes were associated with BW (Table S6). Four growth-related SNPs were located in the intronic region of *Shroom3*. The ion transport-related gene *Nkcc1* and the cytoskeletal, growth, and metabolism-related gene *Mapkap1* were identified near SNP16:1462653.

GO enrichment analysis showed that these candidate genes were significantly enriched in intracellular signaling regulation, cell injury, cell proliferation, and transmembrane transport. Based on KEGG analysis, the growth-related enriched pathways included the PPAR signaling pathway and salivary secretion (Supplementary Figure S1).

### 2.5. Verification of Growth-Related SNPs

Genotyping was conducted for SNP16:3984271 and SNP16:4120214, and growth comparisons were made across individuals with different genotypes. The highest average values for BW, BL, BH, and BT were observed in individuals with CC genotypes of SNP16:3984271 and AA genotypes of SNP16:4120214. The CC genotypes of SNP16:3984271 and AA genotypes of SNP16:4120214 exhibited significant differences compared with the TT genotypes, respectively, in the four growth traits (Supplementary Figure S2).

### 2.6. Joint Analysis of Significant SNP Peak and LD Block

**Figure 4:**
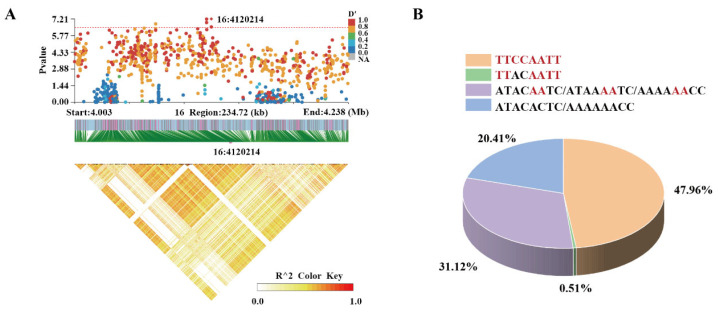
SNP screening and growth-advantageous genotype enrichment. (**A**) Locus zoom plot of a significant SNP peak. (**B**) Statistical analysis of growth-advantageous genotypes. Red indicates the growth-advantageous genotype.

The figure shows all LD SNPs located within the 100 kb region upstream and downstream of SNP 16:4120214. Four significant growth-related SNPs were identified that were in strong LD with SNP16:4120214. All four SNPs were located in *Shroom3* (Figure 4A, Table 2).

The GWAS results also revealed the existence of four SNPs, including the significant SNP peak, within *Shroom3*. Consequently, *Shroom3* was identified as a pivotal gene influencing largemouth bass growth in high-salt environments. The individuals were enriched with most of the four growth-advantageous genotypes (47.96%), with those enriched with three growth-advantageous genotypes representing the lowest proportion. The number of individuals with the most growth-advantageous genotypes is more than twice the number of individuals with no growth-advantageous genotypes. (Figure 4B).

### 2.7. Molecular Characterization and Expression Analysis of Shroom3 in Different Organizations

**Figure 5:**
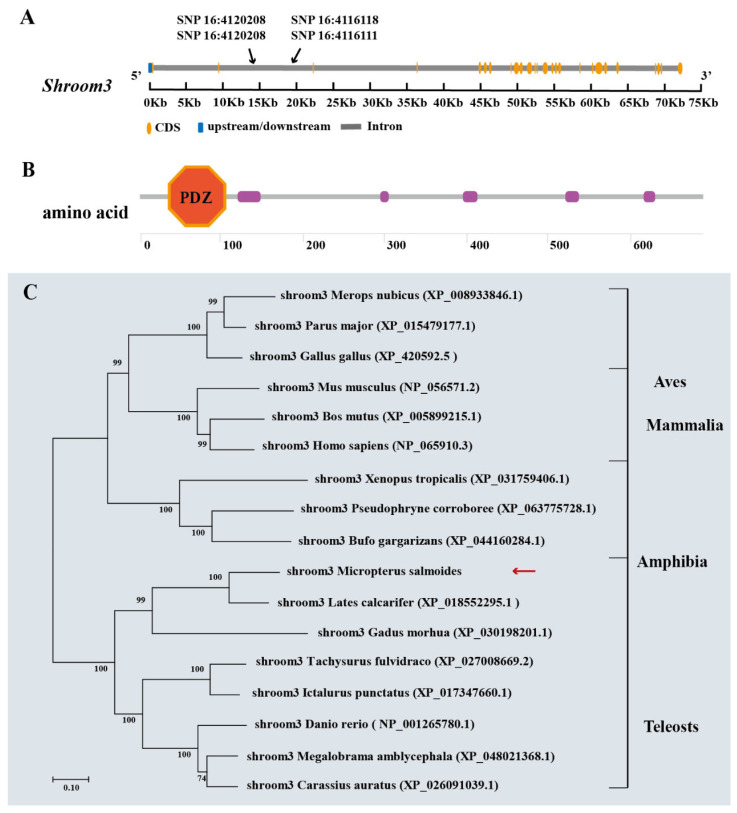
Bioinformatics analysis of *Shroom3.* (**A**) Gene coding sequence and SNP distribution. (**B**) Protein conserved domain prediction. (**C**) Phylogenetic analysis of *Shroom3* from selected species. The red arrows represent the subjects of this study.

*Shroom3* of largemouth bass was 72968 bp long and contained 26 exons. The coding DNA sequence region was 12215 bp long and encoded 2082 amino acids. The N-terminal segment of the protein possessed a conserved PDZ domain (Figure 5A,B). The Shroom3 protein homology between largemouth bass and mandarin fish (*S. chuatsi*), grouper (*Epinephelus. fuscoguttatus*), and large yellow croaker (*Larimichthys. crocea*) were 80.0%, 75.7%, and 72.4%, respectively (Supplementary Figure S3). The phylogenetic tree revealed that largemouth bass and other bony fishes, including Perciformes, Siluriformes, Gadiformes, and Cypriniformes, clustered into one group. Conversely, mammals, birds, and amphibians clustered into another independent clade. This indicated that *Shroom3* was functionally conserved across several taxa (Figure 5C).

**Figure 6:**
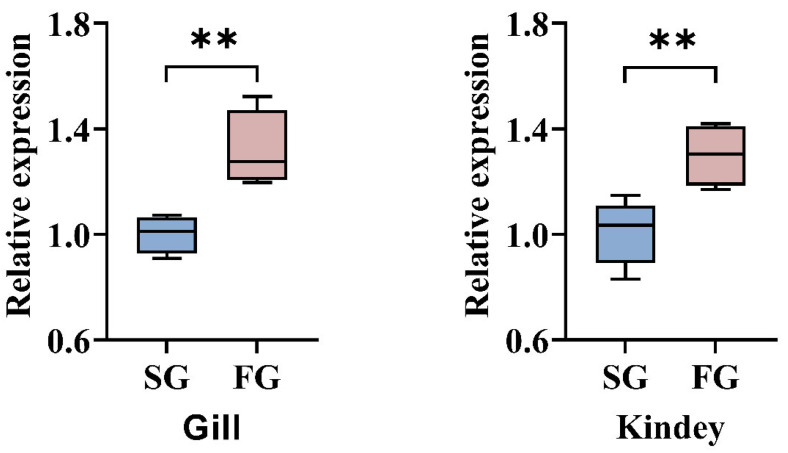
*Shroom3* expression levels of largemouth bass with different numbers of growth-advantageous genotypes. SG, individuals not containing growth-advantageous genotypes; FG, individuals with the four growth-advantageous genotypes. Asterisks indicate significant differences (** *p* < 0.01).

Largemouth bass with the four growth-advantageous genotypes exhibited significant expression differences of *Shroom3* in the gills and kidneys compared with individuals without the growth-advantageous genotypes. The *Shroom3* transcript levels increased in individuals who had the four growth-advantageous genotypes (Figure 6).

## 3. Discussion

The increasing salinization of freshwater seriously threatens the survival of numerous freshwater species [21]. The metabolic activities, tissue structure, and physiological and biochemical indicators of fish are affected when salinity changes [22,23,24]. The largemouth bass is a freshwater fish farmed on a large scale in China. As a result, semi-saline water aquaculture has become an adaptive strategy to cope with the shortage of freshwater. Despite evidence that largemouth bass can tolerate a range of salinities, there has been little research into the effects of osmotic stress on growth regulation [17]. GWAS has significant advantages for locating genomic mutation sites and identifying candidate genes. We established the genetic basis of inter-individual differences in the salt-tolerant and fast-growing traits by genetic analysis of these traits. The findings of this study contribute to the development of molecular markers and the acquisition of superior salt-tolerant and fast-growing strains of largemouth bass.

Heritability and genetic correlations are pivotal parameters that inform the implementation of breeding programs [25]. Narrow-sense heritability quantifies the extent to which genetic factors influence population phenotypes [26]. In this study, narrow-sense heritability for growth traits was calculated based on the proportion of phenotypic variation explained by genome-wide SNPs. The results showed a heritability of 0.53 for BW in largemouth bass, which is higher than that of grass carp (0.42) [27], carp (0.26) [14], and bighead carp (0.20) [28]. The magnitude of heritability is related to an individual’s growth stage and living environment [29,30]. Therefore, reliable heritability estimates are essential for planning an effective largemouth bass breeding program. The present study found strong positive genetic correlations among four growth traits in largemouth bass, with genetic correlations for BL, BW, and BH exceeding 0.9. In summary, largemouth bass have high heritability for growth traits, which are suitable for individual selection. Growth traits also have strong positive genetic correlations, and rapid genetic improvement can be achieved through selection.

Polymorphic SNPs are invaluable tools for studying population genomics and genetics, as they can explain the genetic factors underlying phenotypic differences at the molecular level. Sequence data from high-throughput SNP analysis can be used to construct high-density genetic linkage maps and to produce SNP microarrays, thus facilitating the selection of economically valuable traits [31,32]. Association analysis of SNPs with growth traits has been a widely used approach in fish breeding, and was used in the discovery of multiple growth-associated SNPs in orange-spotted grouper (*Epinephelus coioides*) [33], mandarin fish (*S. chuatsi*) [34], and large yellow croaker (*L. crocea*) [35]. SNPs significantly associated with BW and BL in Chinese longsnout catfish (*Leiocassis. longirostris*) and a significant QTL associated with muscle production in rainbow trout (*O. mykiss*) were centrally mapped on chromosome 16 [36,37]. Similar results were found in this study, and the multiple growth-associated SNPs obtained were also centrally mapped on chromosome 16. It is noteworthy that SNP16:1462653 was simultaneously associated with two growth traits. A single SNP was also identified as being associated with multiple growth traits in olive flounder (*Paralichthys olivaceus*) and golden pompano (*Trachinotus ovatus*) [38,39]. The results indicate that growth traits are quantitative traits that are influenced by multiple genes, and in cases where pleiotropy occurs, a single genetic variant is associated with multiple traits [40].

Ionocytes and ion transport proteins are crucial for maintaining the equilibrium between extracellular and intracellular ion concentrations in fish. The sodium–potassium–chloride cotransporter (Na^+^/K^+^/2Cl^−^ cotransporter, NKCC) is a critical ion transport protein involved in the regulation of osmolality in fish by controlling the entry and exit of Na^+^, K^+^, and Cl^−^ into and out of cells. *Nkcc* expression is closely related to fish growth in a hypertonic environment. Growth hormone injection into Atlantic salmon increased NKCC expression [41]. Additionally, a significant positive correlation was observed between the standardized metabolic rate and *Nkcc* expression in tilapia that were moved from freshwater to seawater [42]. In this study, we found that the active functions of ion transport proteins were significantly enriched. Moreover, *Nkcc1* was annotated near SNP16:1462643. This finding indicates that *Nkcc1* is crucial in maintaining an equilibrium of osmotic pressure and regulating largemouth bass growth in high-salt environments.

*Mapkap1* is related to the cytoskeleton and growth metabolism. It encodes a protein that is an essential component of mTORC2 [43]. The mTOR signaling pathway is at the core of growth regulation. In mammals, mTOR regulates several important biological processes, such as skeletal development, cell proliferation, cell survival, and energy metabolism [44,45]. It was found that mTOR is closely related to fish growth and development. For example, in triploid crucian carp (*Carassius auratus*), mTOR expression was observed to be higher during growth and development, and the growth rate was faster [46]; in grouper juveniles (*E. coioides*), the protein content of the fish was positively correlated with the mTOR mRNA expression level in the muscle [47]. Consequently, this SNP may indirectly regulate *Mapkap1* expression and contribute to regulating largemouth bass growth in high-salt environments.

In addition, when cells respond to external environmental changes (e.g., hypoxic stress and osmotic stress), adaptive regulation is achieved through the mTOR signaling pathway. For example, EMO-treated carp can inhibit the phosphorylation level of mTOR and activate the AMPK/mTOR signaling pathway to enhance autophagy, which protects against acute hypoxia-induced apoptosis [48]; transgenic tilapia (*O. niloticus*) larvae cultured in brackish water showed increased levels of TOR mRNA in the liver compared with those cultured in freshwater, thereby promoting protein synthesis [49]. Therefore, MAPKAP1, as the main mTOR component identified in this study, probably plays a vital part in the growth of largemouth bass by regulating osmoregulation and reducing stress levels.

In addition to the above candidate genes, metabolism-related genes are often used as growth candidates. This study identified fatty acid transporter protein 6 (Slc27a6) and 3-hydroxy-3-methylglutaryl-CoA synthase 1 (Hmgcs1) metabolism-related genes. KEGG analysis showed that the PPAR metabolic pathway was significantly enriched, and *Hmgcs1* and *Slc27a6* were crucial genes in the PPAR signaling pathway. *Slc27a6* is an essential gene in the uptake and deposition of highly unsaturated fatty acid in fish liver tissue [50]. Studies have demonstrated that dietary supplementation of highly unsaturated fatty acids benefits fish growth performance [51,52]. Under low-salinity stress, *Hmgcs1* was involved in adaptation of lipid metabolism of the half-smooth tongue sole to salinity changes [53]. It is important to mention that these candidate genes do not directly regulate largemouth bass growth in high-salinity conditions.

*Shroom3* encodes a cytoskeletal protein with a PDZ structural domain at its N-terminal end, a functional structural domain associated with ion channels [54]. *Shroom3* plays a pivotal role in the functional development of the mammalian kidney [55]. A study revealed that inhibiting *Shroom3* expression in zebrafish (*Danio rerio*) disrupted the glomerular filtration barrier [56]. The metabolic levels of fish are regulated by their kidneys to enable them to adapt to environmental stresses [57,58]. However, exposure to excessive salinity levels can result in the glomerular thylakoid membrane wrinkling and the capillary lumen constricting, which leads to functional inhibition of the filtration process [59]. *Shroom3* expression was relatively increased in the kidney tissue of individuals with more growth-advantageous genotypes in this study. Therefore, *Shroom3* upregulation in hyperosmolar environments may indirectly influence fish growth by maintaining the functional integrity of the kidney.

Further exploration is needed to understand these candidate genes’ biological functions and expression patterns. Additionally, the association between candidate genes and target traits should be assessed along with the overall genetic effects of multiple crucial genes. In the future, the role of these candidate genes in salinity regulation and fish growth will be investigated through gene knockouts.

## 4. Materials and Methods

### 4.1. Statement of Ethics

All experiments followed the Guidelines for the Care and Use of Laboratory Animals in China. The use of experimental fish in this study was approved by the Ethics Committee of the Freshwater Fisheries Research Center of the Chinese Academy of Fisheries Sciences (FFRC, Wuxi, China).

### 4.2. Management and Sample DNA Extraction

Parent candidates were collected from northern subspecies of largemouth bass in 2020. Fingerlings for the study population (F1) were bred by parent candidates in 2023. A total of 500 fish, with an initial average weight of 70.56 ± 3.34 g, were selected for short-term rearing in a freshwater concrete pit (4.7 × 8.3 × 1.3 m) for seven days. For the salinity stress experiment, the salinity was increased to 6‰ at a rate of 3‰/day and subsequently increased to the target salinity of 11‰ at a rate of 1‰/day. The experiment lasted two months, during which the largemouth bass were fed with commercial feed (crude protein ≥48% and crude fat ≥3%) twice per day, at 7:00 and 17:00. The water was replaced twice daily. Water temperature was maintained at 23–25 °C, dissolved oxygen > 5 mg/L, ammonia < 0.02 mg/L, and nitrite < 0.01 mg/L.

The fish were anesthetized with 100 mg/L MS-222 for largemouth bass sedation before sampling [60]. The measurements of body weight (BW), body length (BL), body height (BH), and body thickness (BT) were taken from a sample of 200 arbitrarily selected largemouth bass. Approximately 0.5 g of caudal fin tissue was excised and immediately frozen in liquid nitrogen, and then transferred to a −80 °C refrigerator for storage. Genomic DNA was extracted from the tail fins of largemouth bass using a genomic DNA extraction kit (Vazyme, Nanjing, China) according to the manufacturer’s instructions. The quality of DNA extraction was detected by 0.8% agarose gel electrophoresis, and DNA was quantified by UV spectrophotometer (Thermo Fisher Scientific, Waltham, MA, USA).

### 4.3. Sequencing Library Preparation, Genotyping, and Data Filtering

The sequencing library was prepared using the standard library building procedure of Illumina’s TruSeq DNA PCR-free prep kit (Illumina, Inc., San Diego, CA, USA). The main steps included DNA fragmentation, end repair, junction ligation, PCR amplification to construct the library, and library fragment selection and purification. After quantification of the libraries, the qualified libraries were subjected to 2 × 150 bp double-end sequencing using a NovaSeq 6000 sequencer (Illumina, San Diego, CA, USA).

Raw data were filtered for high-quality data using fastp 0.23.1 and the sliding window method with a 5 bp window size and a 1 bp step size [61]. The bwa 0.7.12-r1039 mem algorithm was used to align the high-quality data obtained after filtering to the largemouth bass reference genome (accession number: ASM2243578v1), and the parameters of the alignment were based on the default parameters of bwa-mem [62]. If multiple paired reads had the same chromosomal coordinates after alignment, only the paired reads with the highest scores were retained. GATK 3.8 was used for SNP detection [63]. The IndelRealigner command in the GATK program was used to align all insertion–deletion mutations near the reads to improve the accuracy of SNP prediction. The obtained SNP loci were further filtered using the UnifiedGenotyper program with the following filtering criteria: (a) Fisher test of strand bias (FS) ≤ 60; (b) HaplotypeScore ≤ 13, mapping quality (MQ) ≥ 40, quality depth (QD) ≥ 2; and (c) ReadPosRankSum ≥ −8.0, MQRankSum > −12.5. The SNP loci were annotated using ANNOVAR 2017 [64].

### 4.4. Population Genetic Structure and Linkage Disequilibrium Analysis

GCTA 1.26.0 was used to analyze the principal components (PCs) of 200 samples using SNP data. The principal component (PCA) plot was drawn using the R package ggplot2 (3.5.1). Gmatrix (v2) was used to calculate a G-value matrix heat map, which was generated using the R package pheatmap (1.0.12) to visualize the kinship between individuals. PopLDdecay 3.42 was used to calculate r^2^ between SNPs to determine the number of markers in linkage disequilibrium (LD). In this study, r^2^ = 1 indicated no recombination at the two sites, r^2^ = 0 indicated no LD or linkage balance, and r^2^ ≥ 0.33 indicated high LD.

### 4.5. Assessment of Genetic Parameters for Growth Traits

Narrow-sense heritability (h^2^) of population growth traits was estimated using the Genomic Relationship Matrix (GRM) calculated with GCTA software [65]. The evaluation of genetic power was based on the single-trait animal model:

y=Xb+Wu+e

$$Var(y)=A\sigma_{g}^{2}+I\sigma_{e}^{2}$$

Here, *y* is the phenotype value vector of each growth trait, such as body length, body height, body thickness, and body weight, *b* is a vector of the first three PCs obtained from principal component analysis, *u* is a vector of SNP effects, with $u\sim N\;(0,I\sigma_{u}^{2}$), *A* is interpreted as the genetic relationship matrix between individuals, *I.* is an n × n identity matrix, and *e* is a vector of residual effects, with $e\sim N\;(0,I\sigma_{e}^{2})$.$\;\sigma_{g}^{2}=N\sigma_{u}^{2}$, with N being the number of SNPs. The formula for narrow-sense heritability was as follows:

$$h^{2}=\frac{\sigma_{g}^{2}}{\sigma_{g}^{2}+\sigma_{e}^{2}}$$

In addition, the software GCTA was used to estimate genetic correlations of four growth traits.

### 4.6. Growth Trait GWAS

The genotype data were filtered before GWAS, and the filtering conditions included the removal of SNPs with a minor allele frequency < 0.05 and deletion rate > 20%. Because GWAS accuracy is affected by the genetic structure of a population, the top three PCs were selected as covariates for GWAS of the four traits recorded using the FarmCPU model of GAPIT 3.4 [66]. The Bonferroni method was used to correct the *p*-value threshold for GWAS to minimize false-positive results. The suggestive threshold was set to 1/N, where N is the number of markers used for association analysis. QQ plots and Manhattan plots were generated using the R package CMplot 3.6.0 to visualize the results [67].

### 4.7. Annotation of Candidate Genes

Based on the LD decay distance when r^2^ decayed to around 0.2, 100 kb upstream and downstream of each SNP above the suggestive threshold were selected as candidate regions significantly associated with the growth trait. Candidate regions were mapped to the reference genome of largemouth bass using the bwa 0.7.12 mem program.

topGO 1.0 was used to perform gene ontology (GO) enrichment analysis [68]. During the analysis, the gene list and gene number of each term were calculated using the candidate region of genes annotated by GO terms to identify GO terms that were significantly enriched in the candidate region of genes compared with the whole genome background. ClusterProfiler 4.6.2 was used to perform Kyoto Encyclopedia of Genes and Genomes (KEGG) enrichment analysis [69]. The candidate genes were annotated by KEGG pathway analysis to identify the gene list and number of genes for each pathway, which facilitated inference of the primary biological functions of the genes (the criterion for significant enrichment was *p* < 0.05).

### 4.8. Verification of Growth-Associated SNPs

A total of 200 fish were arbitrarily selected to extract DNA for genotyping to verify the SNP loci associated with growth potential (obtain from GWAS). We performed NGS-based genotyping for one exonic SNP and the significant SNP peak. The primers and sequence are in the Supplementary Material (Tables S1 and S2). The multiplex PCR amplification conditions consisted of 95 °C for 15 min; 4 cycles of 94 °C for 30 s, 60 °C for 10 min, and 72 °C for 30 s; and 20 cycles of 94 °C for 30 s, 60 °C for 1 min, and 72 °C for 30 s. The PCR products were detected by 3% agarose gel electrophoresis. The qualified products were subjected to NGS, and the results were then analyzed.

### 4.9. LD Block Synthesis and Growth-Advantageous Genotype Statistics

In the 200-tailed validation population, the 100 kb region upstream and downstream of the significant SNP peak was screened for SNPs with high LD (D′ > 0.8) with the initial SNP. The population SNP information was used to obtain haplotype blocks with LDBlockShow 1.40 [70]. High-LD SNPs were analyzed for single-marker associations with growth traits and statistical growth-advantageous genotypes of high-LD SNPs. Individuals enriched with the most growth-advantageous genotypes were used as the fast-growing group (FG), and individuals enriched with the least growth-advantageous genotypes were used as the slow-growing group (SG).

### 4.10. Analysis of Candidate Gene Sequences and Expression Patterns Under Different Salinities

The *Shroom3* DNA and cDNA sequences of largemouth bass were obtained from the sequencing results. GSDS 2.0 (http://gsds.cbi.pku.edu.cn/; accessed on 13 January 2025) was used to visualize the gene structure. The *Shroom3* amino acid sequence was inferred using ORF Finder (https://www.ncbi.nlm.nih.gov/orffinder/; accessed on 13 January 2025). Protein conserved domains were predicted using SMART (http://smart.emblheidelberg.de; accessed on 13 January 2025). The Shroom3 orthologous protein sequences of six other fish species were acquired from the NCBI. DNAMAN (Lynnon BioSoft, San Ramon, CA, USA) was used for multiplex alignment analysis of amino acid sequences from different fishes. Finally, Mega 4.0 was used to construct a phylogenetic tree. The phylogenetic tree was constructed using the neighbor-joining method [71].

Nine fish each were selected from the FG and SG groups for gill and kidney sampling. All samples were lysed with TRIzo1, and RNA was extracted. RNA was reverse transcribed by HiScript^®^ II Q RT SuperMix (Vazyme, Nanjing, China) and the cDNA concentration diluted to 500 ng/μL as a template for fluorescence quantification. qRT-PCR was conducted using gene-specific primers (Table S1), with *β-actin* as the internal control gene. qRT-PCR was conducted using 2× ChamQ Universal SYBR qPCR Master Mix (Vazyme, Nanjing, China), following the manufacturer’s instructions. Amplification conditions were 95 °C for 30 s, followed by 40 cycles of 95 °C for 10 s and 58 °C for 30 s. The gene relative expression levels were assessed using the 2^−∆∆Ct^ method [72], and each reaction was repeated three times.

### 4.11. Statistical Analysis

All data are presented as the mean ± standard deviation. The Shapiro–Wilk test was employed to assess the normality of the data, and the Levene test was used to examine the homogeneity of variances. Significance was inferred by one-way analysis of variance and least significant difference tests. A *t*-test was performed to determine the significance of gene expression differences between groups. The statistical analyses were performed using SPSS 22.0, and *p* < 0.05 was considered statistically significant.

## 5. Conclusions

This study identified 10 growth-related SNPs in largemouth bass stock at 11‰ salinity levels using GWAS. We identified the candidate genes *Nkcc1*, *Mapkap1*, *Shroom3*, *Slc27a6*, and *Hmgcs1*, which are involved in ion transport, growth, development, and cell proliferation. NGS and qRT-PCR analysis revealed that *Shroom3* was an essential gene that influenced largemouth bass growth in a hypertonic environment. In conclusion, these results provide new perspectives on the effects of brackish water aquaculture on largemouth bass growth and provide breeding insights into cultivating salt-tolerant and fast-growing largemouth bass.

## Figures and Tables

**Table 1:** SNPs associated with growth traits in largemouth bass in a high-salinity environment.

Trait	SNP	−Log (*p* Value)	Allele	Position
BH	16:1462653	6.63270	A/T	intergenic
BT	16:1462653	6.98889	A/T	intergenic
	16:3192874	6.74434	T/G	intergenic
	16:3967223	6.50020	A/T	Intronic (*Dcp2*)
	16:3984271	6.89572	C/T	Exonic (*Psmb7*)
	16:4072454	6.77821	T/A	Intronic (*Mfsd14ba*)
	16:4116111	7.19193	G/A	intronic (*Shroom3*)
	16:4116118	6.89294	A/T	intronic (*Shroom3*)
	16:4120208	6.51879	A/T	intronic (*Shroom3*)
	**16:4120214**	**7.20708**	A/T	intronic (*Shroom3*)
BW	16:1964199	6.64524	G/C	Intronic (*Ralgps1*)

Note: Red bold font indicates significant SNP peak among all SNP.

**Table 2:** High-LD SNPs with the significant SNP peak in *Shroom3*.

SNP	16:4102436	16:4155322	16:4173817	16:4116285	16:4110656	16:4121717	16:4124047
REF	G	T	A	T	C	A	T
ALT	A	C	G	A	A	C	C
D’	0.855	0.807	0.856	0.813	0.816	0.826	0.809

Note: Red bold font indicates SNPs significantly associated with four growth traits. REF, reference allele; ALT, alternative allele.

## Data Availability

All data generated or analyzed during this study are included in this published article and its supplementary material files.

## Supplementary Materials

The following supporting information can be downloaded at https://www.mdpi.com/article/10.3390/ijms26051834/s1.
