# Supplementary material for: Genome-Wide Association Study Reveals Growth-Related SNPs and Candidate Genes in Largemouth Bass (Micropterus salmoides) Adapted to Hypertonic Environments
Source: Int J Mol Sci. 2025 Feb 20;26(5):1834. doi: 10.3390/ijms26051834 (PMC11899790; doi:10.3390/ijms26051834)
Supplement: Supplementary file 1 [file ijms-26-01834-s001.zip › Supplementary Material-Figures.pdf]

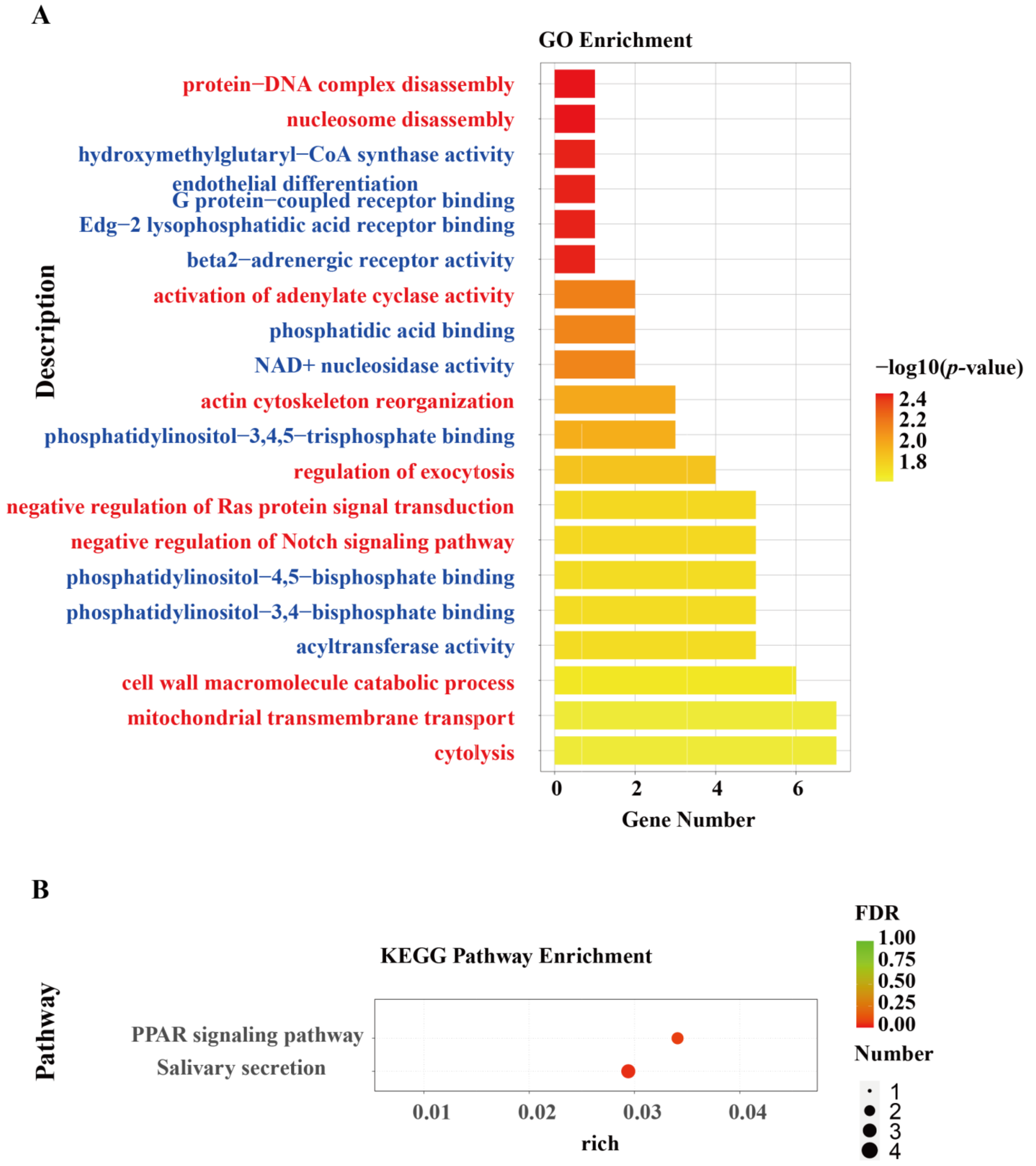

Figure S1. Candidate gene functional analysis (A) GO enrichment analysis for candidate genes. BP: biological process (Red GO terms), MF: molecular function (Blue Go terms). (B) KEGG analysis of candidate genes. The larger the Rich factor, the greater the degree of enrichment, FDR generally takes a value in the range of 0-1, the closer to zero, the more significant the enrichment.

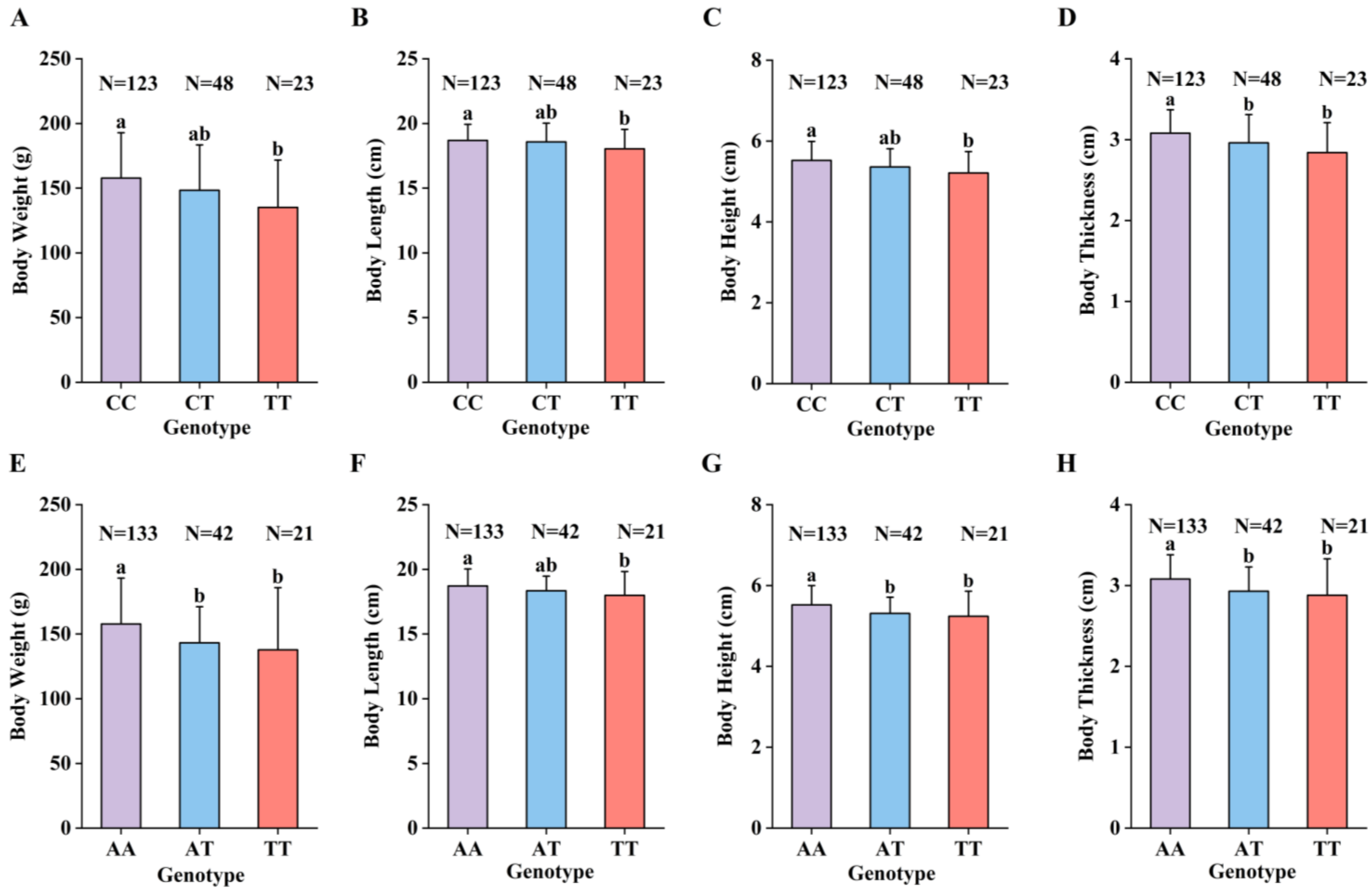

Figure S2. Growth trait statistics of different genotypes for SNP16:3984271 and SNP16:4120214 in largemouth bass. (A-D). For SNP16:3984271; (E-H). For SNP16:4120214. Different lowercase letters indicate significant differences within the group (P < 0.05).

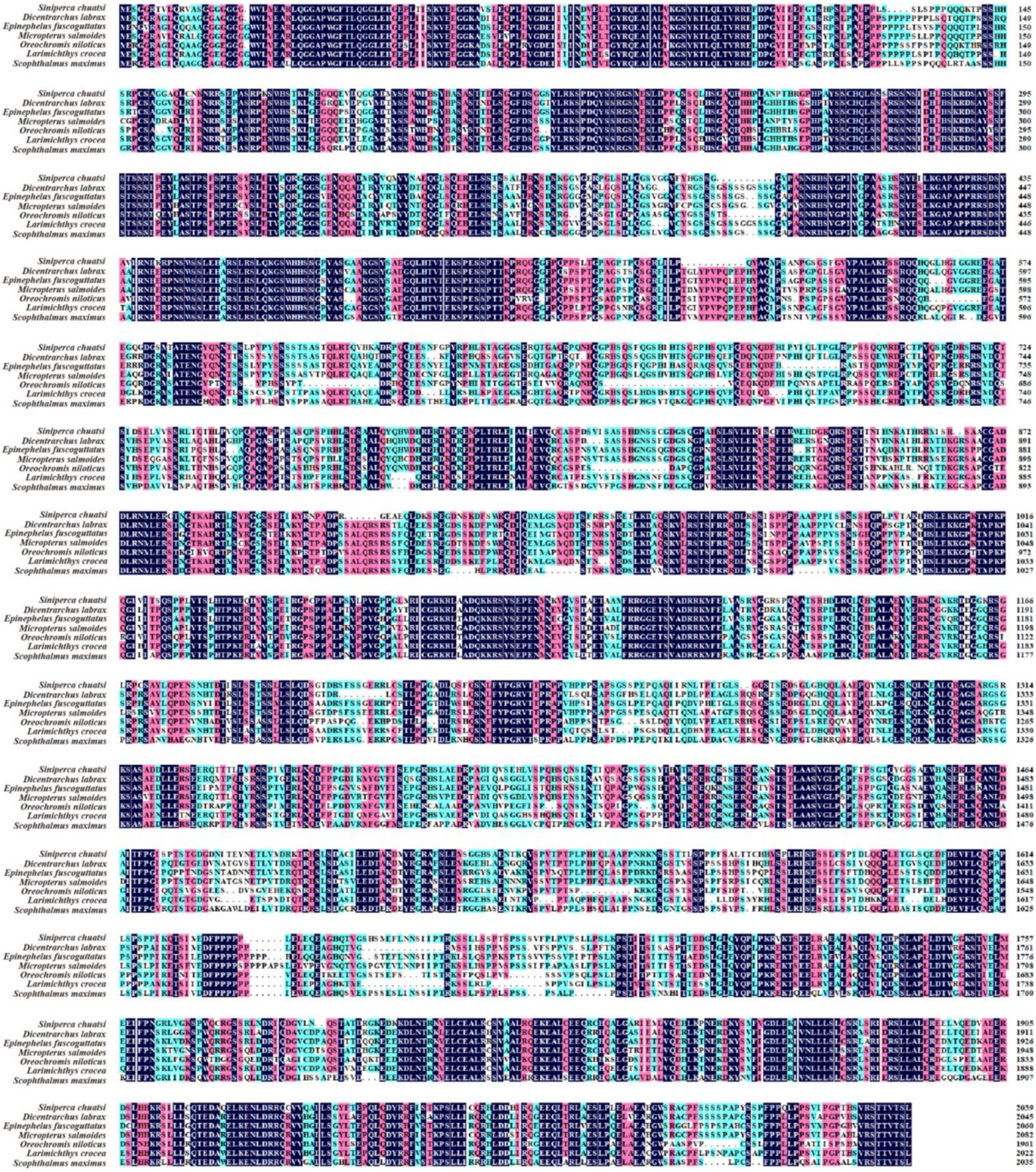

Figure S3. Multiple alignment analysis of *Shooom3* amino acid sequence in different species. Black: Homology=100%; Pink: Homology≥70%; Blue: Homology≥50%.
