# Supplementary material for: Genome-Wide Association Study Reveals Growth-Related SNPs and Candidate Genes in Largemouth Bass (Micropterus salmoides) Adapted to Hypertonic Environments
Source: Int J Mol Sci. 2025 Feb 20;26(5):1834. doi: 10.3390/ijms26051834 (PMC11899790; doi:10.3390/ijms26051834)
Supplement: Supplementary file 1 [file ijms-26-01834-s001.zip › Table S1.pdf]

Table S1

Primers used in this study

Type	SNP	Primer (5'-3')	Product size (bp)
SNP genotyping	16:3984271	F: AACAGATTCATCGACCACATCCAG<br>R: CCTCGGCTACTTGTATTGTTGTATGT	325
	16:4120214	F: TGTATTTAGTGAGAAATGGAAGCC<br>R: GTACTGATGGTGATCGTGATTTAC	225
q-PCR	*Shroom3*	F: CAGAGAACACCCACTGACCC<br>R: GACGTTGGTGGTGCTTTGAC	217
	β-actin	F: ATCGCCGCACTGGTTGTTGAC<br>R: CCTGTTGGCTTTGGGGTTC	336
