# Supplementary material for: Genome-Wide Association Study Reveals Growth-Related SNPs and Candidate Genes in Largemouth Bass (Micropterus salmoides) Adapted to Hypertonic Environments
Source: Int J Mol Sci. 2025 Feb 20;26(5):1834. doi: 10.3390/ijms26051834 (PMC11899790; doi:10.3390/ijms26051834)
Supplement: Supplementary file 1 [file ijms-26-01834-s001.zip › Table S4.pdf]

Table S4

Growth statistics for association analysis of 200 largemouth bass.

Traits	Max	Min	Mean±SD	CV/%
BW/g	251.00	65.70	152.56±36.14	23.69
BL /cm	21.90	14.60	18.59±1.35	7.26
BH/cm	6.60	4.20	5.45±0.46	8.44
BT/cm	3.90	2.20	3.02±0.32	11.26
