# Supplementary material for: Genome-Wide Association Study Reveals Growth-Related SNPs and Candidate Genes in Largemouth Bass (Micropterus salmoides) Adapted to Hypertonic Environments
Source: Int J Mol Sci. 2025 Feb 20;26(5):1834. doi: 10.3390/ijms26051834 (PMC11899790; doi:10.3390/ijms26051834)
Supplement: Supplementary file 1 [file ijms-26-01834-s001.zip › Table S5.pdf]

Table S5

Analysis of heritability (Bold font in the table) and genetic correlation between growth trait.

Trait	BW	BL	BH	BT
BW	**0.527**			
BL	0.971	**0.515**		
BH	0.990	0.975	**0.539**	
BT	0.957	0.880	0.918	**0.468**
